# Effects of infant touch on growth-related indexes of preterm infants

**DOI:** 10.12669/pjms.41.12.12682

**Published:** 2025-12

**Authors:** Man Guo, Qiuying Du, Qingqing Yang, Huichan Yang, Hongbin Zhu

**Affiliations:** 1 Man Guo, Department of Pediatrics, Maternity & Child Care Center of Qinhuangdao, Qinhuangdao 066000, Hebei, China; 2 Qiuying Du, Department of Pediatrics, Maternity & Child Care Center of Qinhuangdao, Qinhuangdao 066000, Hebei, China; 3 Qingqing Yang, Department of Pediatrics, Maternity & Child Care Center of Qinhuangdao, Qinhuangdao 066000, Hebei, China; 4 Huichan Yang, Department of Pediatrics, Maternity & Child Care Center of Qinhuangdao, Qinhuangdao 066000, Hebei, China; 5 Hongbin Zhu, Department of Pediatrics, Maternity & Child Care Center of Qinhuangdao, Qinhuangdao 066000, Hebei, China

**Keywords:** Behavior, Growth and development, Preterm infants, Sleep quality, Touch

## Abstract

**Objective:**

To investigate the effects of infant touch on the growth and development of preterm infants.

**Methodology:**

This was a retrospective study which included sixty-four preterm infants admitted to Maternity & Child Care Center of Qinhuangdao from June 2023 to May 2024 were recruited as study subjects and randomly assigned into the control group (n=32) and the observation group (n=32). The preterm infants in the control group received conventional monitoring measures, while those in the observation group received additional infant touch twice a day for 20 minutes each time for two months on top of the control group.. The physical development, and sleep quality indexes of preterm infants 60 days after birth were statistically compared.

**Results:**

At day 60 of life, the weight and height of the preterm infants in the observation group were markedly higher than those in the control group (*P*<0.05), their levels of muscular, visual, linguistic and emotional development were significantly better than those in the control group(*P*<0.05) and their frequency of nocturnal awakenings and crying duration were notably lower than those in the control group(*P*<0.05). Moreover, infant touch showed no significant effect on head circumference, hearing and sleep quality of preterm infants at day 60 of life(*P*>0.05).

**Conclusion:**

Infant touch boasts of promoting the growth and development of preterm infants in the early stages of life in multiple ways, such as promoting their weight and height growth, vision, language and emotion and enhancing their sleep quality.

## INTRODUCTION

Preterm infants are born at 28-37 weeks gestational age with a birth weight below 2.5kg. They are physiologically immature as a result of the forced early interruption of uterine development. Statistics show that 11.4% of live births are preterm to varying degrees. Preterm birth typically results in low birth weight and neurodevelopmental delay, possibly with complications such as cerebral palsy, respiratory disease and retinopathy, which puts a heavy burden on the body of preterm infants. Preterm birth is one of the major risk factors for infant and child morbidity and mortality.^1^ Studies suggest that preterm infants may be adversely affected in terms of normal growth and neurodevelopment owing to inadequate skin stimulation provided by skin contact with the amniotic fluid and uterine wall;^2^ some of them may even be extremely insecure by the absence of parental contact during guardianship, which is not conducive to their physical and mental healthy development.^3^ In this sense, appropriate external stimulation is of great importance to the growth and development of preterm infants.

The sense of touch is one of the most developed senses in the human body. Tactile and kinesthetic stimulation, such as friction and limb stretching, are frequently used in clinical practice to foster neonatal physical and neurological development. Gentle and soothing touch massage has been proven to efficiently diminish stress hormone levels in newborns,^4^ improve their weight^5^ and sleep quality, relieve pain^6^ and enhance intellectual and motor development.^7^ Considering the difference between preterm infants and their full-term peers in sensory processing and interaction and communication with their parents,^8^ further systematic research is needed to investigate the effects of infant touch on the growth and development of preterm infants. For this reason, 64 preterm infants admitted to our hospital were selected as study subjects for conventional care and touch massage care respectively. We systematically analyzed the role of infant touch on the growth and development of preterm infants through physical development, behavioral development and sleep quality indexes.

## METHODOLOGY

This was a retrospective study. Sixty-four preterm infants admitted to Maternity & Child Care Center of Qinhuangdao from June 2022 to May 2023 were recruited as study subjects and randomly assigned into two groups: the control group(n=32) and the observation group(n=32). Data were retrieved from the hospital information and management system.Collect their various information of all patients. Our study included premature infants with a gestational age of 30-36 weeks, with good general condition at birth, without severe complications or gastrointestinal abnormalities.

### Ethical approval:

The study was approved by the Institutional Ethics Committee of Maternity & Child Care Center of Qinhuangdao (No.: QHDFY-2023041910; Dated: April 19, 2023) and written informed consent was obtained from all guardians.

### Inclusion criteria:

- Preterm infants with a gestational age of 30-36 weeks.
- Those with a birth weight of less than 2.5 kg.
- Those who were born in good condition with neither history of asphyxia nor any complications throughout the observation.
- Those with Apgar score above eight in one minute and five minutes.
- Those families gave informed consent to participate in this study and signed an informed consent form.

### Exclusion criteria:

- Preterm infants with interruption of infant touch during the observation period;
- A birth weight of < 1,000 g
- Those whose families did not cooperate with the investigational procedures.

The preterm infants in the control group received basic examination and monitoring measures, while those in the observation group received gentle infant touch from the first day of life on top of the basic care. In the initial phase, the duration of touch was adjusted to the infant’s adaptation and a frequency of touch twice a day, each lasting 20 minutes, was generalized after the first week of life. Infant touch is typically performed in the early morning and late evening when infants are awake. Prior to infant touch, the caregiver put the infant in a comfortable position such as supine or recumbent with clean hands in a quiet environment with a comfortable temperature. When touching, take the appropriate amount of baby-specific body lotion or emollient oil in the palm was taken, rub it warm and then gently applied it to the infant’s body, gently touching the infant’s head, face, chest, abdomen, back, hands, legs and feet in turn.

During the process, the caregiver should maintain a good mood and patience to observe the infant’s state carefully. In case of abnormalities such as crying or abnormal breathing, the position, intensity and area of touch can be adjusted and if necessary, the touch can be terminated in time to rule out the cause of risk. In this study, infant touch care was provided by healthcare providers during hospitalization and parents were trained in touch skills; after discharge, parents continued to provide touch care at the prescribed frequency. An online healthcare-parent contact group was set up and specialized healthcare providers conducted regular online and offline follow-up visits, answered questions and collected data, both group were folllow up for six month.

### Blinding Procedure:

Parental non-blinded design: Due to the infant touch intervention (requiring active parental participation in skin contact), this study is a non-blinded study. The parents were aware of the grouping situation but were told to avoid communicating the details of the intervention with other subjects. (2)Evaluators were blinded: Neurodevelopmental assessment (NBNA) was performed by neonatologists who were unaware of the grouping situation, and standardized scoring scales were used to reduce measurement bias. (3)Statistical analysts are blinded: In the data analysis stage, the blinding method is adopted, and statistical personnel only come into contact with anonymous grouping codes.

### Observation indexes:

At day 60 of life, physical developmental indexes such as weight, height and head circumference of preterm infants were measured and behavioral developmental indexes such as muscle (angle and duration of head up in a prone position), vision (visual distance), hearing (determination of sound source), language (number of pronounced syllables) and emotion (number of smiles) were counted and sleep quality indexes such as sleep duration, frequency of nocturnal awakenings and crying duration were recorded. Neurodevelopmental assessment was conducted at 40 weeks corrected age using NBNA to evaluate the intervention’s early effects. Subsequent developmental progress was monitored through standard clinical examinations rather than repeat NBNA testing.

### Statistical analysis:

All data were statistically analyzed using the SPSS 22.0 software (SPSS Inc., Chicago, IL, USA). Measurement data in the table were expressed as (χ̄±s) and *t*-test was used for the analysis of differences between groups. Enumeration data were expressed as frequency (%) and the chi-squared (χ^2^) test was used for differences between groups. A *P*-value <0.05 was considered a statistically significant difference.

## RESULTS

Sixty-four preterm infants were included for observation and their basic birth information is shown in Table-I. The results showed no statistically significant difference in male-to-female ratio, gestational age, birth weight, head circumference and birth height (*P*<0.05) between the two groups, which were comparable.

**Table-I:** Comparative analysis of the basic information of the two groups.

	Control group (n=32)	Observation group *(n=32)*	t/χ^2^	P
Gender [cases (%)]			0.063	0.802
Male	18 (65.71)	17 (39.22)		
Female	14 (34.29)	15 (60.78)		
Gestational age (wk., *Χ̅±S*)	34.116±1.400	34.147±1.242	-0.094	0.925
Birth weight (kg, *Χ̅±S*)	2.045±0.214	2.051±0.218	-0.110	0.913
Head circumference (cm, *Χ̅±S*)	32.759±2.445	32.747±2.339	0.020	0.984
Birth height (cm, *Χ̅±S*)	45.308±2.787	45.199±2.905	0.153	0.879

The physical development of the preterm infants of the two groups at day 60 of life is shown in Table-II. The results showed that at day 60 of life, the weight and height of the preterm infants in the observation group were markedly higher than those in the control group (*P*<0.05). The mean head circumference of the preterm infants was slightly higher in the observation group than in the control group, with an insignificant difference (*P*>0.05).

**Table-II:** Comparative analysis of physical development of the two groups at day 60 (*Χ̅±S*)..

	Control group (n=32)	Observation group *(n=32)*	t	P
Weight (kg)	4.639±0.273	4.878±0.252	-3.510	0.001
Head circumference (cm)	35.805±2.504	36.424±2.327	-1.023	0.310
Height (cm)	52.067±2.701	53.467±2.532	-2.140	0.036

The behavioral development of the preterm infants of the two groups at day 60 of life is shown in Table-III. The results showed that the levels of muscular, visual, linguistic and emotional development in the observation group were significantly better than those in the control group, with statistically significant differences (*P*<0.05) and the hearing development demonstrated a tendency to outperform that of the control group (0.05<*P*<0.10).

**Table-III:** Comparative analysis of behavioral development of the two groups at day 60 [cases (%)].

	Control group (n=32)	Observation group *(n=32)*	t/χ^2^	P
Muscle strength Head up 30° in a prone position ≥3s			4.655	0.031
Yes	18 (56.3%)	26 (81.3%)		
No	14 (43.7%)	6 (18.7%)		
Vision Accurate tracking of bright-colored objects at a distance of ≥30cm			6.178	0.013
Yes	21 (65.6%)	30 (93.8%)		
No	11 (34.4%)	2 (6.3%)		
Hearing Determination of sound source			3.475	0.062
Yes	22 (68.8%)	29 (90.6%)		
No	10 (31.2%)	3 (9.4%)		
Language Ability to pronounce ≥3 syllables			4.146	0.042
Yes	15 (46.9%)	23 (71.9%)		
No	17 (53.1%)	9 (28.1%)		
Emotion Frequent smiles back at guardians			5.497	0.019
Yes	16 (50.0%)	25 (78.1%)		
No	16 (50.0%)	7 (21.9%)		

The sleep quality of the preterm infants of the two groups at day 60 of life is shown in Table-IV. The results showed no statistically significant difference in sleep duration between the two groups of preterm infants (*P*>0.05). However, the frequency of nocturnal awakenings and crying duration were notably lower in the observation group than in the control group, with statistically significant differences (*P*<0.05).

**Table-IV:** Comparative analysis of sleep quality of the two groups at day 60 (*Χ̅±S*).

	Control group (n=32)	Observation group *(n=32)*	t	P
Sleep duration (h)	17.028±0.899	17.291±0.817	-1.223	0.226
Frequency of nocturnal awakenings (times)	3.156±0.767	2.469±0.6214	3.941	<0.001
Crying duration (min)	3.619±1.043	2.716±0.7238	4.023	<0.001

## DISCUSSION

The present study indicated a notable contribution of infant touch to the weight and height of preterm infants, proving its favored effect on the early growth and development of preterm infants. Hwu LJ et al.^9^ yielded findings similar to those of our study, who applied touch care to preterm infants with a mean gestational age of 35.1 weeks. The findings indicated a significantly higher weight gain in preterm infants who received touch at weeks 1, 4, 8 and 12 than in those who did not receive touch (*P*<0.05). Further, Lestari KP et al.^10^ conducted touch care twice a week in newborns with a mean birth weight of 2.29 kg. They found that infant touch boosted weight gain in neonates 1-6 months of age with a history of low birth weight (*P*<0.05). More studies have identified a superior mean height of infants after 20 weeks of touch massage compared to the control group, which is consistent with the findings of the present study. A key reason is that touch massage stimulates the intestinal peristalsis of newborns, improves the efficiency of digestion and absorption of nutrients and accelerates the growth and development rate of preterm infants in terms of weight and height. Despite a slight increase in head circumference in the observation group over the control group in this study, touch care did not contribute significantly to the promotion of head circumference. This is probably limited by the lower physical fitness of preterm infants, the slow rate of early neonatal growth and development, or the short duration of the touch cycle. In this regard, further extension of the care cycle is necessary to observe the long-term effects of touch on the development of preterm infants.

Preterm infants are vulnerable because of their inherent physical deficiencies compared to normal newborns. They require meticulous care from doctors, nurses and parents and may even need to be admitted to the neonatal intensive care unit (NICU) for close monitoring. For this reason, relieving the physical burden of preterm infants and promoting their stable and rapid physical development have emerged as the key to care.^11^ Progressively, the positive effects of clinical infant touch on the physical development of newborns have been highlighted. Infant touch is defined as the gentle stimulation of an infant’s body within the normal physiologic range of motion in a systematic application of touch, stroking, rubbing, kneading and stretching.^1^,^12^ It has been indicated that infant touch boosts vagal and gastric activity, elevates serum insulin levels and positively affects neurological and brain development, while at the same time reducing the risk of neonatal sepsis and relieving neonatal stress.^13^-^16^ However, further research is needed to verify the desirable effects of applying infant touch in the clinical care of preterm infants.

Preterm labor impairs neonatal brain perceptual functions and motor abilities, potentially resulting in compromised gross and fine motor skills.^17^ Positive interventions for preterm infants during the prime time of the early postpartum period are of great significance for the behavioral development of preterm infants. Lee HK conducted touch intervention twice a day for 10 consecutive days in preterm infants with gestational age less than 36 weeks, birth weight less than 2kg and no congenital anomalies. The results showed that the vagal tone of preterm infants after the massage was significantly higher than that before the massage, accompanied by a marked enhancement of wakefulness and an increase in motor activity, which suggests that touch massage enhances the ability of preterm infants to respond positively to their environment.^18^ This view is backed up in the present study.

As shown in our study, touch massage greatly facilitates the behavioral development of preterm infants’ muscle, vision, language and emotion (*P*<0.05) and there is a tendency to significantly promote hearing development (0.05<*P*<0.10). This indicates that the touch stimulation during the touch process can be transmitted to the brain of preterm infants through the sensors of the skin; moreover, preterm infants can receive more emotions and information in good interaction with healthcare providers and parents, contributing to their understanding of and response to the external environment, promoting the development of their own neurological and perceptual functions and ultimately accelerating the process of behavioral development.

Sleep troubles after childbirth are a major concern for newborns and their caregivers. Although sleeping for a long time every day, hunger, physical discomfort and environmental stress may induce poor sleep such as multiple nocturnal awakenings and prolonged crying in neonates, which may affect their own growth and development. Therefore, high-quality sleep is one of the primary influences on neonatal growth and development. Juneau AL et al.^19^ conducted a 12-week touch care for neonates between 10 and 14 days of age, with a 30 min touch massage every night. They found that newborns with touch care exhibited dramatically increased melatonin levels (*P*<0.05) and had better sleep-wake cycles compared to the control group. It has also been noted that the number of massages was positively correlated with the total sleep duration of mothers and infants, whilst negatively correlated with the frequency of nocturnal awakenings,^20^ suggesting that infant touch enhances the quality of sleep-in infants.

This study also shows that although infant touch has little effect on sleep duration, it can notably reduce the frequency of nocturnal awakenings and crying duration of preterm infants, suggesting that infant touch improves the sleep quality of preterm infants on the basis of not changing the sleep duration. This is probably because the gentle stimulation of touch massage facilitates the relaxation of the newborn’s body, accelerates their familiarization with the environment, relieves their body of any tension and discomfort they may be experiencing and ultimately boosts their sleep quality as well as normal growth and development.

### Limitations:

The limitations of this study are the short trial period and the fact that only the short-term effects of touch care on the growth and development of preterm infants were investigated. The long-term effects of touch care on the growth and development of preterm infants have not yet been investigated. In this regard, we need to conduct further studies to verify the benefits of this rapid development in childhood and even in adulthood.

## CONCLUSIONS

Infant touch boasts of promoting the growth and development of preterm infants in the early stages of life in multiple ways, such as promoting their weight and height growth, accelerating the process of behavioral development such as muscle, vision, language and emotion and enhancing their sleep quality.

### Authors’ Contributions:

**MG** and **QD:** Carried out the studies and are responsible and accountable for the accuracy or integrity of the work.

**QY** and **HY:** Participated in its design and drafted the article.

**HZ:** Collected the data and performed the analysis. Critical Review.

All authors have read and approved the final manuscript.
